# Recent advances on molecular dynamics-based techniques to address drug membrane permeability with atomistic detail

**DOI:** 10.1016/j.bbadva.2023.100099

**Published:** 2023-08-16

**Authors:** André M.M. Gomes, Paulo J. Costa, Miguel Machuqueiro

**Affiliations:** a BioISI - Instituto de Biossistemas e Ciências Integrativas, Faculdade de Ciências, Universidade de Lisboa, Lisboa, 1749-016, Portugal; b Departamento de Química e Bioquímica, Faculdade de Ciências, Universidade de Lisboa, Lisboa, Portugal; c Faculdade de Farmácia, Universidade de Lisboa, Lisboa, Portugal

**Keywords:** Lipid bilayer, Enhanced sampling, Passive diffusion, Lipophilicity, Constant-pH MD, 0000, 1111

## Abstract

Several factors affect the passive membrane permeation of small molecules, including size, charge, pH, or the presence of specific chemical groups. Understanding these features is paramount to identifying or designing drug candidates with optimal ADMET properties and this can be achieved through experimental/knowledge-based methodologies or using computational approaches. Empirical methods often lack detailed information about the underlying molecular mechanism. In contrast, Molecular Dynamics-based approaches are a powerful strategy, providing an atomistic description of this process. This technique is continuously growing, featuring new related methodologies. In this work, the recent advances in this research area will be discussed.

## Graphical abstract

**Figure:**
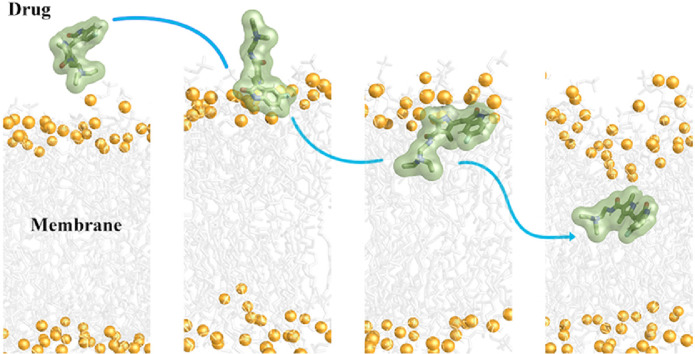

## 1 The role of membrane permeability in drug design

Drug development is a multidisciplinary process with contributions from many different fields of research [1] as it relies on a fine balance between several core parameters that affect all phases in drug discovery up to the final product [2]. Indeed, key parameters such as potency, selectivity, and ADMET properties (absorption, distribution, metabolism, excretion, and toxicity) are paramount for the overall success of drug design [3]. The preferred form for drug administration is oral due to its convenience and cost-effectiveness, and thus, absorption is a major factor for oral bioavailability since molecules need to primarily cross the gastrointestinal tract to reach their target and, consequently, achieve the desired pharmacological effect [4]. As Mannhold et al. stated, “Many of the processes of drug disposition depend on the ability or inability to cross membranes, hence there is a high correlation with measures of lipophilicity” [5].

The molecular transport across membranes can be achieved by different mechanisms, typically categorized into passive or active processes. The most common mechanism for the absorption of small neutral molecules is the passive transcellular diffusion, which comprises the diffusion through the lipid bilayers driven by concentration gradients [4]. In this process, key physicochemical properties (e.g., $pK_{a}$, solubility, lipophilicity), physiological factors (e.g., surface area, pH gradient, transit time), and dosage form are directly associated with the amount of drug absorbed [5]. Despite the apparent complexity of drug absorption, this process can be simplified into fundamental events that control oral drug absorption, such as the drug permeability through the gastrointestinal membrane and its solubility/dissolution in the gastrointestinal sphere [6]. Therefore, the study of passive membrane permeability is paramount not only to understanding elementary biological processes but also to drug discovery and development. In this context, there are several strategies to tackle such a process which can be classified into two major categories, namely experimental and computational or *in silico* approaches. In this mini-review, although also providing an empirical outlook, we mainly focus on computational methods, more specifically on advances in molecular dynamics-based techniques to study drug membrane permeability with atomistic detail, typically covering the last five years of the literature.

## 2 Quantitative measures of lipophilicity

Since the introduction of the Biopharmaceuticals Classification System (BCS) in 1995 [6] and its worldwide spread, the rational drug design and drug product regulatory sciences stepped up into a simpler and more uniform system to define the basic starting point for drug product development. This guidance allowed categorizing drug substances into four groups based on qualitative terms related to aqueous solubility, intestinal membrane permeability, metabolism, and excretion [6], [7], [8]. In this scope, the predominant background for the first two terms mentioned above was based on experimental aqueous solubility data and permeability estimations based on the partition coefficient (log *P*), respectively [7]. The partition coefficient is a term that describes the intrinsic lipophilicity of an organic compound in the absence of ionization or dissociation [5] and it is defined by the equilibrium concentration ratio of a compound in the organic (most commonly, 1-octanol) and aqueous phases (Eq. (1)).

(1) $$P=\frac{{\left[drug\right]}_{organic}}{{\left[drug\right]}_{aqueous}}$$

After obtaining an estimate of the lipophilicity, this property can be decomposed as a sum of contributions from fragments such as individual functional groups. Thus, the impact of the addition of a new substituent group into a molecule could in principle be predicted.

Considering that a large number of drugs comprise ionizable groups [9], a further ionization equilibrium is needed to describe lipophilicity. This equilibrium relies on the pH of the aqueous phase and thus depends on the $pK_{a}$ of the ionizable group [5]. Notice however that the $pK_{a}$ values of drugs at the water/membrane interface will most likely be different from the expected $pK_{a}$ value in solution [10], [11], bearing important implications for membrane permeability since under normal conditions, only drugs in their neutral form are able to cross into the organic phase. The global contribution of both equilibrium states, neutral and ionized, of the drug ratio across phases is described by a single term referred to as the distribution coefficient (log *D*) [12]. For a monoprotic acid (HA), the distribution coefficient is given by Eq. (2)

(2) $$D=\frac{{\left[HA\right]}_{organic}}{{\left[HA\right]}_{aqueous}{\left[A^{-}\right]}_{aqueous}}$$

where [HA] and [A^-^] represent the concentrations of the acid in its neutral and dissociated states, respectively. Moreover, the ionization of a compound in water is given by its dissociation constant (K_d_, Eq. (3)).

(3) $$K_{d}=\frac{\left[H^{+}\right]\left[A^{-}\right]}{\left[HA\right]}$$

The combination of the previous equations results in the pH distribution relationship (Eq. (4))

(4) $$\log D=\log P-\log(1+10^{pH-pK_{a}})$$

which relates both log *P* and log *D*. Thus, both log *P* and log *D* emerge as an approach to quantify lipophilicity and provided medicinal chemists with a descriptor thought to be correlated with membrane permeability.

## 3 Knowledge-based methods and experimental models

With the decisive role of lipophilicity well-established for the potential bioavailability of a drug candidate, other guidelines thrive to describe drug-likeness and acceptable oral exposure. One of the greater examples in medicinal chemistry is Lipinski’s “Rule of 5” (RO5) [13]. Indeed, RO5 intended to align the chemical behavior into worthwhile compounds, taking into consideration their oral bioavailability and states that, in general, drug candidates fulfill the following criteria: molecular weight ≤ 500 Da, log *P* ≤ 5, hydrogen bond donors ≤ 5, and hydrogen bond acceptors ≤ 10 [13]. Although the predictive character of RO5 is reasonable, this benchmark is only useful in discarding compounds with a high probability of presenting poor oral activity. Additionally, fulfilling the RO5 criteria has a lack of meaning concerning the specific functional features of drugs and does not ensure that a molecule is drug-like. On the other hand, there are several examples of established drug molecules that do not obey the RO5.

Several research groups attempted to define the chemical space occupied by drug-like molecules on knowledge built from drug databases. The idea is that drugs tend to hold similar values for certain properties (descriptors). Hence, these descriptors could be used to define a point in a multi-dimensional space that intrinsically characterizes each molecule [14]. Moreover, other simple methods reinforced the previously mentioned idea. For instance, functional group filters supported the identification of undesired moieties due to their chemical reactivity and metabolic unreliability. Alongside these methods, quantitative structure-activity relationships (QSAR) approaches came into play. These are suited as predictive tools in drug discovery and associated fields, elucidating the influence of different molecular properties on specific events. Classical QSAR studies may include several properties, such as lipophilicity and polarizability or certain structural features, aiming at describing their individual impact on biological endpoints, including permeability [15].

**Fig. 1:**
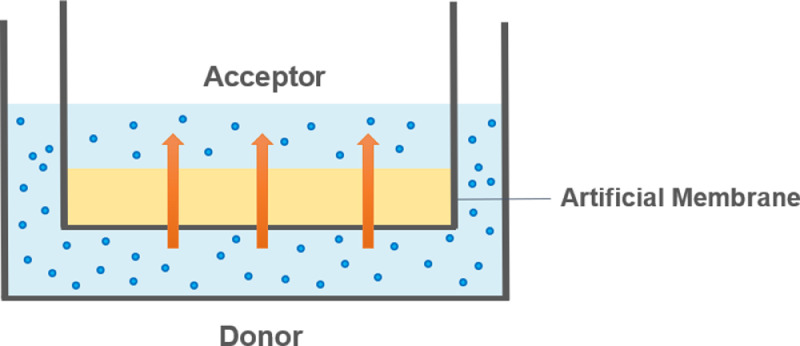
Schematic representation of the PAMPA model, displaying the molecular movement of passive diffusion across a thin microfilter (synthetic membrane) separating two aqueous compartments.

Later on, researchers developed new experimental methods to address this purpose, the most popular one being the parallel artificial membrane permeability assay (PAMPA). PAMPA is a low-cost alternative to cellular models such as the remarkable Caco-2 and MDCK models, for screening the permeability of research compounds in the early stages of drug development. This assay performs permeability measures across an artificial membrane, being an *in vitro* model for passive diffusion (Fig. 1) and providing a better predictivity for oral absorption and blood-brain barrier (BBB) penetration than log *P* calculations or measurements [16]. Thus, its popularity has risen progressively alongside Caco-2 models as experimental approaches to studying passive membrane permeability.

Despite the applicability and convenience of knowledge-based methods and experimental models, these have some limitations. Indeed, atomistic details (e.g., formation of noncovalent interactions) and membrane complexity can be major constraints to the predictability and interpretability of such models when applied to unknown compounds. In fact, different types of bilayers have a diverse range of lipid tails, differing on chain lengths and saturation [17]. This variability influences the permeation of drugs in distinct regions of the cellular membrane. According to Sharifian et al. “diffusion of hydrophilic compounds through a single membrane barrier is the rate-limiting step, while for lipophilic compounds, the membrane may act as both a barrier and a sink” [18]. Thus, the desorption from the membrane into the acceptor compartment might also be a rate-limiting step in overall permeation [19]. Additionally, measuring log *P* values can also be challenging for lipophilic compounds, while this solubility-based strategy does not consider the kinetics, which is more challenging to obtain experimentally [20]. Alternatively, Caco-2 assays could lead to permeation overestimation or underestimation for specific compounds (e.g., efflux substrates, esters, amides, and acids) [21]. Furthermore, in *in vitro* permeability experiments, the unstirred water layer (UWL) also contributes to the total resistance of the permeation barrier [22], [23]. This is a concentration gradient formed between the bulk solution and the intended permeation barrier and reduces the permeability of the tested compounds. The limitations on these experimental methodologies are such that it prompted a clear distinction between *in vivo* membrane permeability and the apparent permeability (P${}_{app}$) values obtained *in vitro*. Although PAMPA may be found as the most balanced model (high benefit-cost), this and the other mentioned approaches lack information about the underlying molecular mechanisms, providing slightly to no insight into the biophysics of membrane permeation at the atomic level.

## 4 Molecular dynamics simulations

The permeation of small molecules through lipid bilayers is a phenomenon that can be directly observed in molecular dynamics (MD) simulations on the nano- and microseconds timescale [20]. Succinctly, this *in silico* approach predicts how forces act on every atom (particle) in a molecular system as a function of time through the calculation of derivatives of potentials [24] using a simplified model of the underlying physics regulating atomic interactions. Newton’s laws of motion are solved iteratively for every particle in the system over several time steps resulting in a trajectory that describes, with full atomistic detail, the dynamics of the molecular system in the simulation time window. The mentioned forces, acting on every atom, result from contributions from bonded and non-bonded interactions between atoms. In this framework, the covalent bonds and the atomic angles are simplified into virtual springs, and dihedral angles are described using periodic functions for bond rotation. Non-bonded interactions proceed from van der Waals interactions, typically described by a Lennard-Jones potential, and from electrostatic interactions, expressed by Coulomb’s law [24], [25].

Together, the equations and underlying parameters are referred to as molecular mechanics “force fields” due to the fact they assemble the contributions of the various atomic forces that shape molecular dynamics [26], [27], [28]. Force fields are usually parameterized and adjusted to reproduce experimental data and/or energies and geometries proceeding from quantum mechanics calculations at a high level of theory. Several force fields are commonly used in MD simulations and they can be categorized as fixed-charge or polarizable force fields. In a classical fixed-charge force field the polarization is usually not modeled explicitly (i.e., the effective partial charges do not change depending on conformation and environment) [29]. The choice of the force field is crucial since its accuracy has a significant impact on the reliability of results. The most popular are the classical fixed-charge ones such as AMBER [26], CHARMM [27], and GROMOS [28], which have been continuously improved over the years.

Despite the complexity of biological membranes, their interactions with other molecules can be studied using MD in model systems. This allows us to systematically investigate the effect of different environmental factors (e.g., lipid composition, pH, ionic strength, level of hydration or temperature) [10], [30], but also the potential formation of noncovalent interactions on membrane permeability [31]. The most common membrane models intend to mimic simplified versions of mammalian plasma membranes which are composed of different lipid structures, namely cholesterol and neutral (zwitterionic) phospholipids like 1-palmitoyl-2-oleoyl-sn-glycero-3-phosphocholine (POPC), 1,2-dipalmitoyl-sn-glycero-3-phosphocholine (DPPC), or 1,2-dioleoyl-sn-glycero-3-phosphocholine (DOPC). However, the complexity of these models is often increased with the addition of anionic lipids to study mitochondria [32], [33].

Using MD data, passive membrane permeability was initially studied using the homogeneous solubility-diffusivity model, based on Fick’s law of diffusion. Later on, the inhomogeneous solubility-diffusion model (ISDM) was developed, which comprises the heterogeneous nature of lipid bilayers, being the gold standard to calculate membrane permeability coefficients [34]. The ISDM derives from the steady-state flux and assumes equilibrium across the membrane, allowing the variation of the diffusion constant and the free energy of the membrane interior [24], [34]. The potential of mean force (PMF), referred to as *W(z)*, and the local diffusivity coefficient, *D(z)*, can be collected from MD simulations data and mathematically related to the resistivity, *R,* and permeability, *P*, using Eq. (5)

(5) $$R=\frac{1}{P}=\int_{z_{1}}^{z_{2}}\frac{exp[\beta W(z)]}{D(z)}dz$$

where β refers to the beta thermodynamic term (β = 1/$k_{B}$*T*), and *z* is a collective variable describing the relative position of the solute along the transmembrane axis. The integration limits, *z_1_* and *z_2_*, are points along this axis on opposing sides of the membrane in the water phase. As mentioned before, both terms *W(z)* and *D(z)* can be estimated from MD simulations results [19], [34].

For this premise to be valid, all *z* positions of the solute need to be well sampled [34]. Inadequate sampling impairs the description of functional features of the system and stems from the rough energy landscapes, with many local minima separated by high-energy barriers, which regulate the biomolecular motion [35]. Due to the character of Boltzmann sampling, conventional MD is not always ideal for sampling the entire configurational space via direct simulations. Indeed, despite all its features and success, conventional MD also presents some limitations, especially concerning sampling issues. Thus, more advanced methodologies rooted in MD simulations have been developed in the past decade to overcome these obstacles. Some examples comprehend umbrella sampling (US) [36], adaptive biasing force (ABF) [37], metadynamics (MT) [38], and the Wang-Landau algorithm [39]. Methods can also be carried out with multiple replicas (RE) or walkers (MW) to further enhance sampling [24], [34], [35]. In the next section, some relevant advances in the usage of MD simulations for studying passive membrane permeability are discussed.

## 5 Recent MD-based approaches for membrane permeability

In order to estimate the BBB permeability of 26 small-molecule compounds through DOPC and POPC lipid-membrane models, Thai et al. [40] performed a study using steered molecular dynamics (SMD). This method allows the computation of the nonequilibrium work, $W_{neq}$, produced by pulling the compounds through the membrane. Employing the CHARMM and GROMOS force fields, the authors devised a qualitative correlation between $W_{neq}$ required to pull a compound through the bilayer and the experimental BBB permeability values. These promising results were obtained for both membrane models (DOPC and POPC) and different pulling speeds, independently of the choice of force field. This study, by using atomistic simulations, was also able to provide information regarding the role of hydrogen bonds (associated with low permeability) and energetic barriers during their pulling. Since it is computationally cheap, this method can be seen as a prescreening tool for large datasets of compounds. On the other hand, it did not provide the free-energy profile of the lipid bilayer crossing of the compounds which could be obtained using the Jarzynski Equality. Indeed, also in 2020, Noh and co-workers [41] compared SMD along with the Jarzynski Equality (JE-SMD), with Umbrella sampling (US) for computing free-energy profiles of aromatic substrates through phospholipid bilayers. The JE-SMD could in principle provide an advantage over US because the equilibrium free energy is evaluated from an exponential average of $W_{neq}$ taken from rapid puling non-equilibrium trajectories, thus reducing the computational cost. The simulations were carried out using a coarse-grained (CG) version of a DOPC lipid bilayer and the General Amber Force Field (GAFF) to model toluene and phenylalanine molecules. For both aromatic substrates, the JE-SMD approach presented an insufficient sampling convergence of the bilayer environment and is dependent on the characteristics of the substrate. Furthermore, JE-SMD significantly disrupts the orientation of the substrate and hence hydrogen bond formation, considering the phenylalanine profiles. Therefore, the authors concluded that US remains the more viable to obtain free-energy profiles from MD simulations.

**Fig. 2:**
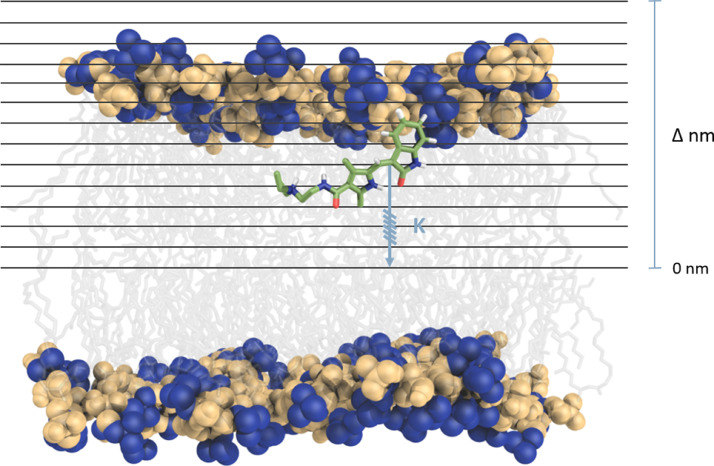
Representation of an Umbrella sampling (US) scheme presenting the respective windows, and applied to a POPC membrane model. The US windows references are illustrated by the horizontal lines shown across the system and, in this case, with the same spacing from each other. The arrow represents the distance to the membrane center which is usually used as the reference and k is the harmonic potential constant. The lipid head groups are shown as blue (cholines) and yellow (phosphates) spheres. (For interpretation of the references to colour in this figure legend, the reader is referred to the web version of this article.)

As it could be hinted above, US simulations are a hallmark in the study of the permeation mechanism of small molecules and the most established technique to obtain the potential of mean force (PMF) along a reaction coordinate [42]. This straightforward strategy restrains the solute to discrete sections along one (or more) reaction coordinates, referred to as “windows” (Fig. 2) using a biasing potential, which is often harmonic due to their simplicity. Hence, the intermediate steps between two thermodynamic states are covered by a series of windows that are subsequently combined using different methods, such as the weighted histogram analysis method (WHAM) or umbrella integration [24], [36]. US has been employed over the past years to enhance sampling and is sometimes combined with other methods in a hybrid approach. In 2021, Faulkner and Leeuw [17] used US to address membrane permeability of an established drug molecule (fentanyl) along with three analogs in a variety of simple phospholipid membrane models (POPC, DOPC, DMPC, and DPPC), employing the ISDM to calculate the permeability values. The results showed that the primary resistance to permeation was observed at the lipid head group due to the chemical features of the molecules, which display a partially charged, polar, and hydrophobic nature. Moreover, the DMPC lipid bilayer provided the best correlation with experimental data from PAMPA and Caco-2 permeability studies. The authors concluded that employing US simulations provides atomistic insight into drug permeation across different regions of the bilayer, producing accurate permeability coefficients which could be used in the design of new fentanyl-based drugs.

More recently, Sousa et al. [43] applied MD simulations with US to explore the effect of hydrophobicity on the passive permeation of inhibitors of the bacterial (*P. aeruginosa*) PqsD enzyme across a membrane model. The solutes were parameterized using GAFF and permeability coefficients were calculated employing both ISDM and free-energy ranking (ΔG_ranking_) models. The ΔG_ranking_ model uses the difference between the maximum and minimum of the free-energy profile for membrane permeation, resulting in better correspondence with the experimental results [44] thus highlighting possible shortcomings of the ISDM for specific systems. The six compounds, with a range of increasing lipophilicities, displayed comparable permeabilities, indicating that in this case, permeability is not strongly affected by differences in hydrophobicity. Moreover, detailed analysis of the molecular orientation along the permeation pathway evidenced divergences between this group of molecules, suggesting a correlation between a favorable orientation pattern and an increased *in bacterio* activity.

As mentioned before, US simulations can be combined with other enhanced sampling techniques such as replica exchange (REUS) to improve the sampling without the addition of substantial computational costs. REUS is similar to US but allows neighboring windows to exchange conformations based on the Metropolis criterion [36], [45]. A study reported by Pokhrel and Maibaum [45] in 2018 intended to compare different methods to calculate the free energy of membrane permeation also addressing the sampling limitations due to strong headgroup-solute interactions. They considered the translocation of three prototype molecules (arginine, alanine, and water) and a sodium ion through DOPC lipid bilayers using MT, US, and REUS. The authors found that all three methods are similar for neutral permeants, whereas, for polar and charged molecules, that is not the case due to electrostatic interactions between lipid headgroups and the solute. Highly polar molecules can affect the relaxation time of the system, which is related to a hysteresis-like behavior, and mislead free-energy calculations. Overall, this effect could only be overcome with REUS calculations, supporting its robustness in evaluating free-energy profiles of membrane permeation or detecting sampling deficiencies by analyzing the exchange pattern between replicas.

Also to tackle sampling issues, in 2022 Harada and co-workers [46] developed two methods with a weak dependency on external biases, only requiring the selection of initial structures and their conformational resampling. These two rare-event sampling methods work together in a hybrid conformational search approach using the parallel cascade selection MD (PaCS-MD) and the outlier flooding method (OFLOOD), both combined with a Markov state model (MSM) construction. Firstly, PaCS-MD generates the initial membrane permeation paths of a compound and afterward, OFLOOD expands the unsampled conformational area around the initial paths. In the end, the obtained trajectories were employed to build MSMs leading to more correct free-energy profile calculations and membrane permeability coefficient estimations. Overall, the results obtained for seven compounds qualitatively correlate with the experimental data, suggesting that this approach is a successful alternative to external bias-dependent methods to achieve sufficient conformational search.

More recently, a new affordable methodology to estimate PMF profiles of bulkier drug-like molecules in membrane models, named MemCros, was developed [47]. The Accelerated Weight Histogram (AWH) method was applied to compute the permeability values of a set of 12 xenobiotics exhibiting a wide variety of chemical scaffolds with pharmacological relevance. The main advantage of the AWH method relies on its fast convergence, which is exponential during the initial stage, and asymptotic afterward. This specificity allows the rapid determination of PMF profiles with low dependency on the input parameters related to the molecule properties. Additionally, it provides a picture of all intermolecular interactions, with atomic resolution, by describing in detail the molecule orientations along the permeation pathway. Overall, the computational cost to obtain a converged estimate of permeability coefficients with MemCross was generally lower than other enhanced sampling methods, such as ABF or US, which require multi-μs simulations for each replica. Nevertheless, it should be noted that other hybrid techniques, e.g., the combination of well-tempered metadynamics and the extended system adaptive biasing force (WTM-eABF) can improve the convergence rate, reduce the dependence on the simulation parameters and significantly lower the computational cost of the permeability coefficients calculations [48], [49].

**Fig. 3:**
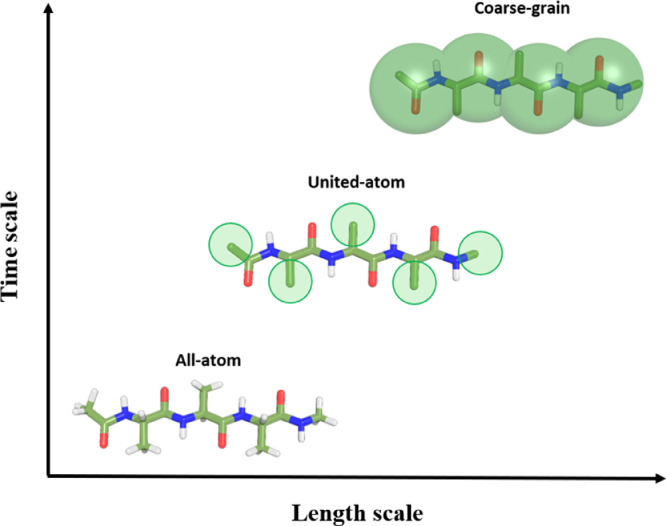
Schematic representation of the molecular structure of a capped trialanine using three different atomistic levels. In the united-atom representation, all apolar hydrogen atoms are collapsed into their carbon atom. The GROMOS force field family is an example of this level of representation [11], [28]. In the Martini force field family, the alanine residues are coarse-grained and represented as single beads [50], [51].

Due to the complexity of large biological systems, classical MD with all-atom (or united-atom) FFs can still present some limitations, namely in system sizes (nm) and time scales (ns to μs) that are computationally feasible. To tackle this issue coarse-grain (CG) molecular dynamics, which is a method that relies on assembling groups of atoms to form “beads” (Fig. 3), can be used. The MARTINI force field, which is a hallmark in CG simulations, was initially designed for studying biological membranes [50], [51], and later extended to further complex biomolecular networks and different coarse-grained levels. This strategy allows faster and much longer simulations at the expense of losing atomistic details. In this scope, recently, Menichetti et al. [52] performed high-throughput coarse-grain (HTCG) simulations addressing the permeability of over 500k compounds comprising organic small molecules (ranging from 30 to 160 Da), deriving a permeability surface based on two molecular descriptors, namely, bulk partitioning free energy and $pK_{a}$. The authors showed a plain connection between specific chemical groups, including acidic, basic, and zwitterionic compounds, and the resulting permeability coefficients calculated using ISDM. Later, CG simulations combined with US simulations were applied to cover 105 molecules, each one represented by two CG beads, to calculate membrane permeability across six different phospholipid membranes and to draw free-energy profiles. Altogether, they ended up building a dataset correlating chemical structures with drug permeability [53].

In order to improve the accuracy and convergence of drug permeation simulations, Ayidin et al. [54] performed a study combining MD with transition-tempered MT to analyze the permeation mechanism of trimethoprim through a multicomponent membrane. They showed that collective variables obtained from a machine learning (ML) algorithm, which embed critical “slow” degrees of freedom that mediate membrane permeation, improve performance and substantially accelerate the convergence of PMF calculations. The work focused on the permeation across a pure POPC membrane and POPC with the addition of cholesterol. The incorporation of cholesterol increases bilayer thickness and also the width and height of the free-energy barrier due to a condensing effect (decreased area per lipid). The algorithm, named time-structure based Independent Component Analysis (tICA), also allowed to capture the subtle effect of cholesterol which leads to increased resistance to permeation in the lipid head group region, which is not observable using canonical collective variables.

Since an ionizable molecule might neutralize at the water-membrane interface before entry [11], hence becoming more permeable, the state-of-the-art methodology to deal with pH effects in a dynamic process is the so-called constant-pH MD (CpHMD). It samples both the charge states of the solute ionizable sites and their conformational dynamics. There are different strategies for introducing these methods in classical MD such as stochastic Monte-Carlo (MC) based approaches and empirical-valence-bond approaches, known as λ-dynamics [55]. On stochastic CpHMD methods, the protonation of each titrable residue is a discrete variable and pH is treated as one of the external thermodynamic parameters in MD simulations. Here, the new states of the ionizable solute can be obtained from Poisson–Boltzmann/Monte-Carlo (PB/MC) calculations [56]. Alternatively, λ-dynamics relies on simulations using the λ-variable to represent the extent of protonation for each ionizable site [57], [58]. In the past few years, CpHMD was applied to study the charge fluctuation of molecules in a wide range of biological systems for all different purposes, including in the membrane permeation context. Indeed, in 2019 Yue et al. [59] applied hybrid-solvent CpHMD along with US free-energy surface analysis to study the permeation of the weak base propranolol (PPL) through a POPC lipid bilayer. The simulations were performed using the CHARMM force field and the permeation results were based on the solubility-diffusion model from PMF values and calculated local diffusivity. The simulations indicated that PPL dynamically neutralizes at the lipid-tail interface, dramatically influencing the permeation free-energy landscape, which is commonly neglected by the conventional simulations and may explain the overestimation of the assigned intrinsic permeability. Finally, the authors were able to obtain PPL passive diffusion as a function of pH, whose values were qualitatively consistent with those determined experimentally by PAMPA.

## 6 Conclusions

In brief, the study of passive membrane permeability can provide crucial insights into vital biological events and naturally affects the drug design process. Several strategies have been developed over the years to address this subject. The main methodologies can be categorized into experimental and computational approaches and both have their own advantages and limitations. Experimental methods, such as PAMPA, may seem a reasonable option to accurately estimate permeability values and infer structure-based relationships. In contrast, Molecular Dynamics-based approaches can be found at a higher level considering the atomistic detail that they can assure. Indeed, *in silico* methods display an extensive potential, not only for their advantages in terms of speed and easiness compared to the experimental perspective but also for the continuous increase in the computational power at our disposal. The estimation of membrane permeability coefficients is now easily achieved but the molecular basis behind its mechanism is still often elusive, as in the case of the effect of protonation changes. Increasing the realism of the models is therefore paramount for a fundamental understanding of the whole process.

## Declaration of Competing Interest

The authors declare that they have no known competing financial interests or personal relationships that could have appeared to influence the work reported in this paper.

## Data Availability

No data was used for the research described in the article.
